# Multi-Sip Time–Intensity Evaluation of Retronasal Aroma after Swallowing Oolong Tea Beverage

**DOI:** 10.3390/foods7110177

**Published:** 2018-10-25

**Authors:** Naomi Gotow, Takanobu Omata, Masaaki Uchida, Naoyuki Matsuzaki, Sadaki Takata, Ippei Hagiwara, Tatsu Kobayakawa

**Affiliations:** 1 Human Technology Research Institute, National Institute of Advanced Industrial Science and Technology (AIST), Tsukuba Central 6, 1-1-1 Higashi, Tsukuba, Ibaraki 305-8566, Japan; nao-gotow@aist.go.jp; 2 NTT Data Institute of Management Consulting, Inc., JA Kyosai Bldg., 10th Fl., Hirakawacho 2-chome, Chiyoda-ku, Tokyo 102-0093, Japan; Takanobu.Omata@sony.com (T.O.); hagiwaraip01@keieiken.co.jp (I.H.); 3 Suntory Global Innovation Center Limited, 1-1-1 Wakayamadai, Shimamoto-cho, Mishima-gun, Osaka 618-8503, Japan; Masaaki_Uchida@suntory.co.jp (M.U.); Naoyuki_Matsuzaki@suntory.co.jp (N.M.); 4 Graduate School of Human Sciences, Osaka Shoin Women’s University, 4-2-26 Hishiyanishi, Higashiosaka, Osaka 577-8550, Japan; takata.sadaki@osaka-shoin.ac.jp

**Keywords:** multi-sip, time–intensity, retronasal aroma, oolong tea beverage, consumption experience, warm-up sample

## Abstract

In most cases, a meal cannot be finished with a single bite and sip. During eating and drinking, consumers receive dynamic food perceptions from sensory attributes in foods. Thus, we performed multi-sip time–intensity (TI) evaluation of sensory attribute. In each of ten trials, the participant evaluated continuously the intensity of retronasal aroma for 60 s after swallowing oolong tea. We compared the TI parameters (*I*_max_: maximum intensity, *T*_max_: time point at which intensity reached the maximum value, *AUC*: area under the TI curve, *D*_plateau_: duration between the first and last time points with values exceeding 90% of the maximum intensity, *R*_inc_: rate of intensity increase between the first time points with values exceeding 5% and 90% of the maximum intensity, and *R*_dec_: rate of intensity decrease between the last time points with values exceeding 5% and 90% of the maximum intensity) and TI curves among the ten trials, and approximated each TI curve with an exponential model. Some TI parameters (*I*_max_, *T*_max_, *AUC*, and *R*_inc_) differed significantly between the first and subsequent trials. The TI curve was significantly lower in the first trial than in the subsequent trials, and TI curve during the time from starting the evaluation to reaching maximum intensity was significantly lower in the second trial than in the subsequent trials. The time constant of the fitted exponential function revealed that the decay of retronasal aroma intensity was slightly faster in the second through fourth trials than in the first and the fifth through tenth trials. These results indicate that olfaction might be more perceptive while consumers sip a cup of the beverage.

## 1. Introduction

### 1.1. Measurement of Temporal Changes in Sensory Attributes

Temporal changes in sensory attributes perceived during eating and drinking provide dynamic food perception to the consumer [1]. The most common method for measuring temporal changes in sensory attributes of food is time−intensity (TI) evaluation [2]. Researchers can obtain more perceptual information that changes with time by performing TI evaluations of sensory attributes rather than single-point evaluation [3]. Previous studies performed TI evaluations of retronasal aroma of foods: skim milk aroma of ice cream [4], vanilla aroma of ice cream [5], fruit aroma of ice cream [6], strawberry aroma of ice cream [7], mint aroma of chewing gum [8,9], cinnamon aroma of chewing gum [10], and meat aroma of pork pâté [11].

In general, TI evaluation is the measurement of the intensity of a single sensory attribute that changes over time in reaction to a single exposure to a stimulus [12]. Dual-attribute TI evaluation [9,13] and multi-attribute TI evaluation [14] have also been proposed, but these methods are not widely applied due to the large burden on the participants and the enormous amount of time required for the experiments [15,16]. The temporal dominance of sensations (TDS) task, in which the participant directs their attention simultaneously to multiple sensory attributes in a single trial, was developed recently [17]. In the TDS task, the temporal change of each sensory attribute is obtained by recoding the most dominant attribute (i.e., the most impressive attribute, but not necessarily the strongest attribute) that changes over the course of time. Several studies employed the TDS task using beverages such as coffee [18,19,20], blackcurrant squashes [21], red wine [22,23], white wine [24,25], and vodka [26].

TDS requires little training for participants [15]. Consequently, TDS is a very simple task that even untrained consumers can perform [27]. For example, consumer panelists have performed TDS using several foods such as chocolate [28], strawberry [29], fish sticks [30], and wine with and without cheese [31]. On the other hand, conventionally, TI evaluation has been performed for trained panelists [12,32,33]. In other words, many researchers reported that it was difficult to perform TI evaluation for untrained panelists. Gotow and colleagues [34] developed a new evaluation system for TI evaluation by untrained panelists. In the conventional TI evaluation system, a lever, a rotary knob, a joystick, and a computer mouse were used as a reaction device, and the output from response device was visually fed back onto a computer screen as movement of a cursor or indicator along the scale [35]. On the other hand, in the TI evaluation system developed by Gotow and colleagues, a load cell connected to a spring, a string, and a metal ring was used as response device. The output from the response device was not only visually fed back onto a computer screen as movement of an indicator along the scale, but also kinetically fed back to the participant’s index finger that was operating the ring. Such dual feedback was expected to improve the performance of participants who had not undergone special training for sensory evaluation. Additionally, previous studies [36,37] reported that when a participant was presented with a taste solution containing an odorant, they paid attention to their tongue, and it became more difficult to detect or identify the olfactory element rather than the gustatory element. Based on these studies, Gotow and colleagues devised a screen that displayed instructions, so that the participant’s attention was directed to different parts of their body in the taste quality and retronasal evaluation sessions. More specifically, with reference to the task in which the participant was asked to report the part of the anatomy that perceived sensory attributes such as vanilla aroma [38,39], the relevant part of the anatomy was displayed on the screen, using an illustration of the sagittal plane of the head with the name of the part (e.g., “on the tongue” in the taste quality evaluation session, and “in the throat” in the retronasal aroma evaluation session). In accordance with previous studies [40,41], each TI curve obtained from participants was approximated with an exponential function, and then correlation coefficients between the actual and theoretical values were calculated. The results indicated that a correlation coefficient of 0.8 or more was observed in about 90% of all TI curves, and that TI curves differed significantly between taste quality and retronasal aroma. Based on these results, Gotow and colleagues concluded that they succeeded in developing a system by which an untrained panelist can easily and precisely perform TI evaluation of the sensory attributes of food. Using this evaluation system, untrained panelists performed TI evaluation of bitterness and retronasal aroma of black coffee beverage without sugar [34,42,43], as well as sweetness of sweetened coffee beverage with milk and sweetened water solution [44].

### 1.2. Multi-Sip Sensory Evaluation

The amounts of consumption of various foods enable us to infer the dietary habits of each consumer, i.e., their consumption experience over the relatively long-term. On the other hand, consumers repeat short-term consumption experiences during everyday meals, such as breakfast, lunch, and dinner. In other words, it is possible to regard actions in which a consumer sips one cup of beverage until the cup is empty as a short-term consumption experience. When a consumer drinks a whole cup of a beverage, it is rare for them to gulp it with a single sip. Since perception changes through repetition of the sipping action, multi-sip evaluation allows researchers to acquire more reliable data, and obtain a deeper understanding of food perception, than can be acquired in a single-sip evaluation [45,46].

Oolong tea is so familiar to Japanese consumers that the amount produced is reported in public statistics [47]. The consumption of oolong tea in Japan in 2017 was 11,042 tons, about 10% of the total consumption of all types of tea [48]. Additionally, according to data reported by the Japan Soft Drink Association, the production of oolong tea beverages in Japan in 2017 was 632,800 kL, about 10% of the total production of all types of tea beverage [49]. Thus, oolong tea is one of the most popular teas, and it has a characteristic aroma [50]. The aroma of oolong tea consists mainly of nerolidol, jasmine lactone, methyl jasmonate, and indole, and it can be characterized as elegant floral aroma with a dried fruit note [51]. The sensory qualities of oolong tea depend on aroma, as well as other sensory attributes such as sweetness, umami, and astringency [52]. Some studies reported that the intensity of aroma of oolong tea was affected by the region where the tea leaves were produced [50], the semi-fermentation time of the tea leaves [53,54], and the type of water used to brew the tea [55].

Multi-sip TDS tasks have the potential to deepen understanding of food perception as a consumer drinks a whole cup of a beverage [45,56,57,58]. Zorn and colleagues [59] performed a study with the TDS task, using four orange juices to which different sweeteners (sucrose, sucralose, thaumatin, and stevia) were added. The TDS tasks, of 20 s per trial, were performed in three consecutive trials. For each sample and each trial, Zorn and colleagues constructed TDS curves for six sensory attributes (sweetness, sourness, bitterness, astringency, orange flavor, and off-flavor). When they compared TDS curves between samples, the dominant ratio of sweetness in sample to which sucrose was added indicated temporal change similar to that of dominant ratio of sweetness in sample to which sucralose was added. In two samples containing thaumatin and stevia, dominant ratios of sourness and bitterness increased with repetition of the trials. These results implied that multi-sip sensory evaluation might enable specification of differences between samples.

Some previous studies performed multi-sip single-point evaluation of taste qualities, and these studies reported that intensity decreased gradually with the repetition of trials [60,61]. For example, Schiffman and colleagues [62] performed multi-sip single-point evaluation using water, 0.27 mM tannic acid solution, and 1.36 mM tannic acid solution. Six sweeteners at four concentrations, another six sweeteners at three concentrations, and the remaining sweeteners at two concentrations were added to each solution, yielding a total 46 sweet solutions. Solutions containing sweeteners at four concentrations had intensity equivalent to 3%, 6%, 9%, and 12% sucrose solutions (in the case of three concentrations, 3%, 6%, and 9%; two concentrations, 3% and 6%). Participants transferred the presented sample into the oral cavity for 5 s, and then spat out it. Immediately after spitting, they evaluated the intensity of sweetness. For each solution, they repeated this procedure in four consecutive trials at 30 s intervals. The results revealed that the intensity of sweetness decreased gradually with the repetition of trials. Thus, for taste quality, some previous studies have employed multi-sip single-point evaluation, but we are aware of no study employing multi-sip TI evaluation. For retronasal aroma, we find neither multi-sip single-point evaluation report nor multi-sip TI evaluation.

In this study, we performed multi-sip TI evaluation of retronasal aroma using oolong tea beverage. To investigate how the perceptual sensitivity of retronasal aroma of oolong tea beverage changed while a participant sipped the beverage, we compared TI parameters and TI curves of retronasal aroma among multiple trials. Based on the study of Gotow and colleagues [42], we hypothesized that perceptual sensitivity of retronasal aroma might improve because the opportunities for the participant to perceive retronasal aroma increase while they sip the beverage. On the other hand, by analogy to previous studies for multi-sip single-point intensity evaluation [60,61,62], we also hypothesized that perceptual sensitivity of retronasal aroma might decrease because the participant adapts to the retronasal aroma as they sip the beverage.

## 2. Material and Methods

### 2.1. Participants

This study was conducted in accordance with the revised version of the Declaration of Helsinki. All procedures in this study were approved by the ethical committee for ergonomic experiments of the National Institute of Advanced Industrial Science and Technology, Japan. When we recruited participants, we made it clear to potential volunteers that oolong tea beverages and salt-free crackers could be used as experimental materials, and that individuals with allergies against any ingredients of these products could not volunteer for the experiment. Before starting the experiment, we reconfirmed that no participant had allergies against the ingredients of the experimental materials. Furthermore, we informed participants of their right to cease participation even after their initial agreement to participate. Informed written consent was acquired from all participants. Twenty-five volunteers (11 female and 14 male) between the ages of 20 and 54 years old (average age ± standard deviation = 26.12 ± 9.57 years old) participated in the experiments. Participants received a reward for participation in this experiment.

### 2.2. Materials

We used 350 mL of plastic-bottled oolong tea beverage (“Suntory *kuro* oolong tea *kaoru* jasmine”, Suntory Beverage and Food Limited, Tokyo, Japan), which does not contain sugar or milk. Salt-free cracker and mineral water were used to clean the participant’s oral cavity [26,63,64]. We opened each package of salt-free cracker (“Premium non-salt topping”, Yamazaki Nabisco, Tokyo, Japan), and mineral water (“Suntory *minami* Alps *no ten-nen sui*”, Suntory Beverage and Food Limited) one hour before the start of experiment. The salt-free cracker was cut to a size of 2 cm × 2 cm, and one piece of cracker was served in a paper candy cup. Mineral water (10 mL) was measured using a macropipette and poured into a paper cup (capacity 90 mL, Part number SM-90-3, Tokan Kogyo, Tokyo, Japan). On the table (width 89.5 × depth 44.5 × high 64 cm) on which the TI system described below was placed, we arranged a plastic-bottled oolong tea beverage with an unbroken seal, two paper candy cups with cracker, two paper cups with mineral water, and a transparent polypropylene cup (“Dispo cup premium clear”, capacity 100 mL, division 10 mL, AS One Corporation, Osaka, Japan) for measuring oolong tea. The plastic bottle containing the oolong tea beverage had a polyethylene commercial label that provided brand information to consumers. We presented oolong tea beverage and mineral water at room temperature (approximately 24 °C).

### 2.3. TI Evaluation System

**Figure 1:**
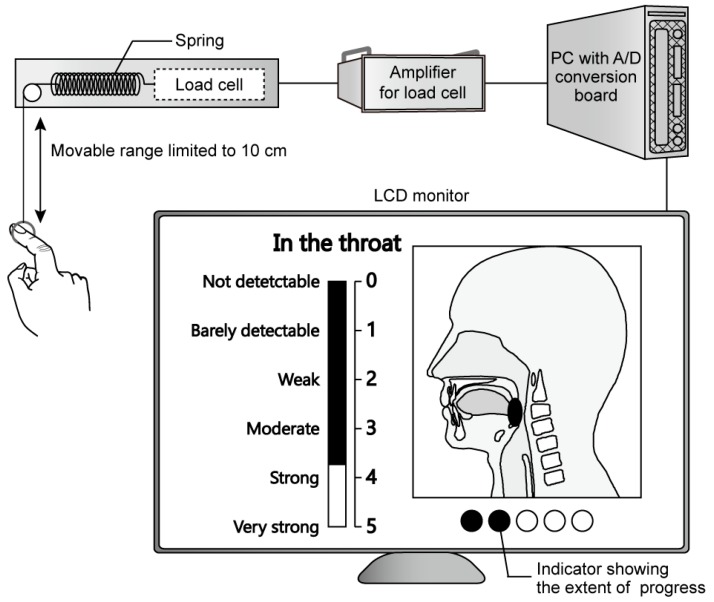
Outline of the TI evaluation system. PC: personal computer; A/D: analog-to-digital; LCD: liquid crystal display.

As shown in Figure 1, in order to perform TI evaluation of retronasal aroma of oolong tea beverage, we used an evaluation system in which a steel ring with a diameter of 2 cm was connected by a string to a spring removed from a spring balance (maximum weighing capacity 0.2 kg; Part number ST-02, AS ONE Corporation, Osaka, Japan). The range of movement of this ring was limited to 10 cm by a stopper (left upper devices drawn in Figure 1). The value of intensity increased as the participant pulled the ring more strongly with their finger. If the participant did not apply force to the ring, it was pulled back by spring tension, and the value of intensity decreased. A six-point magnitude scale (0: “not detectable”, 1: “barely detectable”, 2: “week”, 3: “easily detectable”, 4: “strong”, 5: “very strong”) was used to evaluate intensity [65]. When the ring was located at the original position, the value of intensity indicated “not detectable (0)”. When the ring was pulled until it was blocked by the stopper, the value of intensity indicated “very strong (5)”. The position of the ring, synonymous with spring tension, was measured using a load cell (maximum load 5 N; Part number DTU-5N, Imada, Toyohashi, Japan), with output expressed as voltage. After the output voltage was amplified, it was recorded by a personal computer (PC) through an analog-to-digital (A/D) conversion board (Part number PEX-234104, Interface, Hiroshima, Japan) at a frequency of 1 kHz. To provide visual feedback, the value of intensity was displayed to the participants as a black bar drawn on a six-point magnitude scale on a liquid crystal display (LCD) monitor (screen size, 10.4 inch; part number QT-1003P-AV-TP, Quixun Products, Tokyo, Japan) placed 35 cm in front of the participant. In order to prevent fatigue, the participant was instructed to put their arms on the elbow rest of the chair throughout the TI evaluation. In addition, we previously verified the reliability and validity of the TI evaluation using this system [34,42].

Participants evaluated intensity by operating a pull-ring, which was a component of the evaluation system. The movable range of the ring was limited to 10 cm by a stopper. Positional information of the ring, synonymous with spring tension, was measured by a load cell, with output expressed as voltage. After the output voltage was amplified, it was recorded by a PC through an A/D conversion board at a frequency of 1 kHz. To provide visual feedback in real time, the value of intensity was displayed on an LCD monitor as a black bar on a six-point magnitude scale (0: “not detectable” to 5: “very strong”). Furthermore, to inform the participant of the time remaining in the evaluation, an indicator of the extent of progress was shown on the screen.

### 2.4. Procedure

Participants were expected to perform TI evaluation on the basis of concepts related to aroma and intensity formed through consumption experiences in daily life. Therefore, participants did not receive special training in sensory evaluation. Each participant was asked to perform TI evaluation of retronasal aroma after swallowing oolong tea beverage, in total of ten trials. All instructions were displayed on the LCD monitor placed in front of participant. Gotow and colleagues [42] who developed the TI evaluation system used in this study, reported that participants could easily and precisely perform TI evaluation of sensory attributes of food following a single training trial, which provided an explanation of the evaluation method. In this study, we suspected that the single training trial might have some influence on the evaluation in the main trials, even if the sample presented to participants differed between the training and main trials. Therefore, we did not arrange training trials for our participants. Instead, before starting the first trial, the participant confirmed the instructions with the experimenter by watching a screen, and experienced the operation of the ring.

In the first screen of sequential trials, we instructed the participant to evaluate continuously intensity of retronasal aroma in the throat after swallowing the oolong tea beverage over tens of seconds. In the same screen, referring to previous studies [38,39] in which participants reported which part of their anatomy they used to perceived specific sensory attributes (e.g., some participants replied that they perceived vanilla aroma in their mouth), we instructed the participant regarding the part of the anatomy to which they should direct their attention (i.e., “in the throat”), using an illustration of the sagittal plane of the head with the name of the part labeled (a display drawn in Figure 1). Next, the participant placed a cracker into their mouth to clean the oral cavity, and continued masticating it for 15 s before the screen was switched. At that time, the participant swallowed the cracker remaining in their oral cavity, and then held 10 mL of mineral water in their mouth. After they transferred the water into the oral cavity, they swallowed it. The number of trails was displayed on the screen. In order to prevent as much as possible the aromatic substances contained in oolong tea beverage from volatilizing, the participant opened the cap of the plastic bottle immediately before starting the TI evaluation for each trial, and then poured 10 mL of oolong tea beverage into a cup with divisions. After measuring the oolong tea beverage, they closed the cap. Next, we counted down 5 s [61,62] before the screen instructed the participant to swallow. Before the countdown reached 0 s, the participant took 10 mL of tea beverage in their mouth, which they held without swallowing, and then placed the index finger of their right hand into the ring of the TI evaluation system. The participant swallowed the oolong tea beverage in their mouth at the same time that the countdown reached 0 s, and that the screen showed visual feedback about intensity. Incidentally, in everyday life, consumers do not clean the oral cavity every time they take a sip of beverage. Therefore, in order to unify the conditions in the oral cavity among participants while following the normal practice in daily life, participants cleaned the oral cavity only before starting the first trial.

Participants evaluated intensity over 60 s for each trial. We instructed each participant to demonstrate their intensity of retronasal aroma by freely operating the pull-ring component of the evaluation system. We did not tell the participants the length of the evaluation time (i.e., how long they were to evaluate intensity). Instead, in order to inform the participant of the time remaining in a trial of evaluation, we displayed an indicator on the screen showing the extent of progress. Participants did not rest between trials. The interval from the end of TI evaluation in a given trial to the start of TI evaluation in the subsequent trial was about 30 s. After finishing the tenth trial (i.e., final trial), the participant cleaned the oral cavity using clacker and mineral water, as they had before starting the first trial.

### 2.5. Analysis

#### 2.5.1. Comparison of TI Parameters among Trials

**Figure 2:**
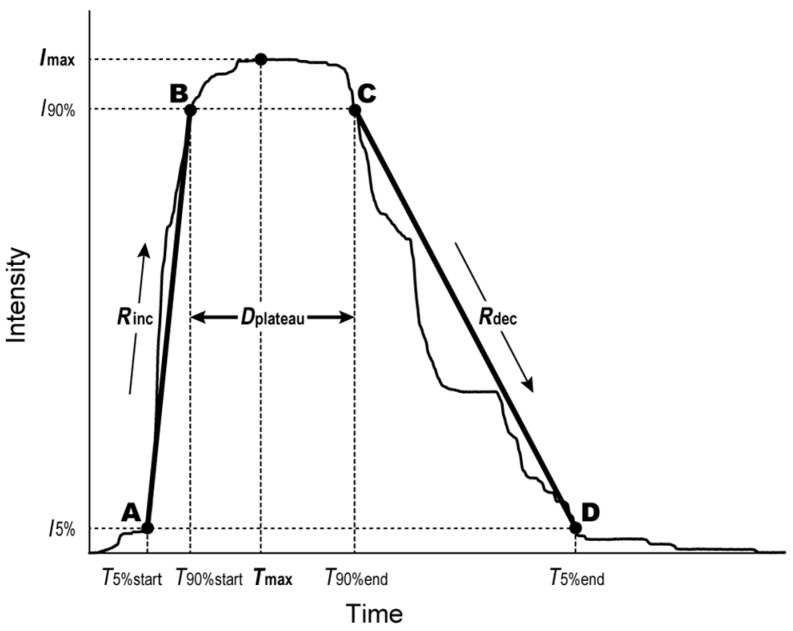
Calculation model for TI parameter. *I*_max_: maximum intensity; *T*_max_: time point at which intensity reached the maximum value; *D*_plateau_: duration between *T*_90%start_ and *T*_90%end_; *R*_inc_: rate of intensity increase between *T*_5%start_ and *T*_90%start_; *R*_dec_: rate of intensity decrease between *T*_90%end_ and *T*_5%end_.

For each TI curve obtained from participants, six TI parameters (maximum intensity (*I*_max_), time point at which intensity reached maximum value (*T*_max_), area under the TI curve (*AUC*), duration of maximum intensity (*D*_plateau_), rate of intensity increase between the time point at which sensation to stimulus was first perceived and *T*_max_ (*R*_inc_), the rate of intensity decrease between *T*_max_ and the time point at which sensation to stimulus was extinct (*R*_dec_)) were calculated. Based on the trapezoidal model of Lallemand and colleagues [6], shown in Figure 2, four points (*A*, *B*, *C*, and *D*) were determined on the TI curve. *A* (*T*_5%start_, *I*_5%_) and *B* (*T*_90%start_, *I*_90%_) were the first points with values exceeding 5% and 90% of the maximum intensity, respectively. *C* (*T*_90%end_, *I*_90%_) and *D* (*T*_5%end_, *I*_5%_) were the last points with values exceeding 90% and 5% of the maximum intensity, respectively. Incidentally, when an evaluation value did not decrease to 5% of the maximum intensity until the end of evaluation after reaching the maximum intensity, the end point of the TI curve was regarded as *D*. *AUC* is the area under the TI curve between *T*_5%start_ and *T*_5%end_. *D*_plateau_ is the duration between *T*_90%start_ and *T*_90%end_. *R*_inc_ is the rate of intensity increase between *T*_5%start_ and *T*_90%start_. *R*_dec_ is the rate of intensity decrease between *T*_90%end_ and *T*_5%end_.

To determine whether the values of TI parameters differed among ten trials, we performed one-way repeated measures analysis of variance (ANOVA) for each parameter, with the trial number as an the inter-subject factor. Simple effects tests were conducted based on the significance of results obtained with ANOVA. Incidentally, for one participant, because the maximum intensity was displayed simultaneously with the start of evaluation, *A* and *B* could not be mathematically identified in multiple trials. Additionally, for four participants, because the evaluation values of TI curves did not decrease to 90% of the maximum intensity until the end of evaluation after reaching the maximum intensity, *C* and *D* could not be identified in one or more trials. Therefore, these five participants were excluded from analysis of TI parameters.

For each TI curve obtained from participants, TI parameters were calculated. *I*_max_ represents maximum intensity, and *T*_max_ represents the time point at which intensity reached the maximum value. Based on the trapezoidal model of Lallemand and colleagues [6], four points (*A*, *B*, *C*, and *D*) were determined on the TI curve. A (*T*_5%start_, *I*_5%_) and B (*T*_90% start_, *I*_90%_) are the first points with values exceeding 5% and 90% of the maximum intensity, respectively. *C* (*T*_90%end_, *I*_90%_) and *D* (*T*_5%end_, *I*_5%_) are the last points with values exceeding 90% and 5% of the maximum intensity, respectively. *AUC* is the area under the TI curve between *T*_5%start_ and *T*_5%end_. *D*_plateau_ is the duration between *T*_90%start_ and *T*_90%end_. *R*_inc_ is the rate of intensity increase between *T*_5%start_ and *T*_90%start_. *R*_dec_ is the rate of intensity decrease between *T*_90%end_ and *T*_5%end_.

#### 2.5.2. Comparison of TI Curves among Trials

In this analysis, we regarded the time when the screen was switched to visual feedback of intensity as the starting point of the TI evaluation (i.e., 0 s). We divided the period from 0 s to 60 s after swallowing into 30 windows of 2 s each, and calculated the average intensity in each time window. We conducted statistical analysis using these average values.

To investigate whether TI curves differ among ten trials, we performed two-way repeated measures ANOVA for the average intensity in each time window, with trial number and time as within-subject factors. Simple effects tests were conducted based on the significance of results obtained with ANOVA.

#### 2.5.3. Approximation of the TI Curve

**Figure 3:**
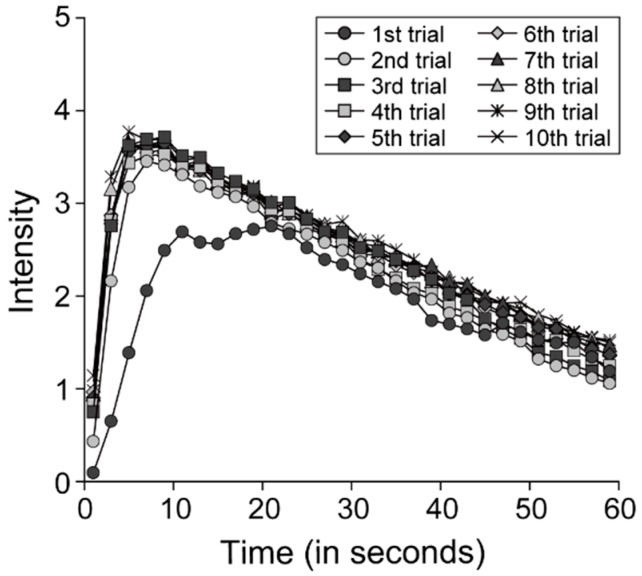
Inter-participant average of TI curves of retronasal aroma.

To more closely examine the temporal change in retronasal aroma intensity after reaching the maximum intensity, we calculated the fitted function for each TI curve obtained from participants. In a previous study [42], the inter-participant average of the TI curve was approximated with the exponential function y=A×exp(−Bt), where *y* is intensity, *A* is a coefficient, *B* is the time constant, and *t* is time (in seconds). Based on that study, with reference to the inter-participant average of the TI curve shown in Figure 3, we used this function to approximate the retronasal aroma intensity in time windows from the time of maximum intensity (i.e., time window with median value of 11 s) to the end of the evaluation, for every TI curve. The time windows to be approximated were determined with reference to the inter-participant average of the TI curve shown in Figure 3. Moreover, for every TI curve, we set *A* and *B* to minimize the root-mean-square error between this function and the TI curve, using the nonlinear method of a generalized reduced gradient. Furthermore, to qualitatively demonstrate the goodness of fit of the exponential model, we calculated Pearson’s product–moment correlation coefficients between the actual values (TI curve) and the theoretical values (fitted exponential function). Larger values for this correlation coefficient indicated that the shape of the TI curve was more similar to the fitted exponential function.

To determine whether the coefficients, time constants, and goodness of fit differed among the ten trials, we performed one-way repeated measures ANOVA for each parameter, with trial number as a within-subject factor. Multiple comparisons by the Ryan method were conducted based on the significance of results obtained with ANOVA.

We used SPSS 10.0 J (SPSS Japan, Tokyo, Japan) for statistical analysis throughout this study, and *p* values less than 0.05 were considered statistically significant. We used the solver function of Microsoft Office Excel 2010 (Microsoft Japan, Tokyo, Japan) to calculate the fitted exponential functions.

## 3. Results

### 3.1. Comparison of TI Parameters among Trials

The values of TI parameters in each trial are shown in Table 1. One-way repeated measures ANOVA for each TI parameter revealed a significant main effect of trial number for *I*_max_ (*F* (9, 171) = 4.64, *p* < 0.001), *T*_max_ (*F* (9, 171) = 5.14, *p* < 0.001), *AUC* (*F* (9, 171) = 5.16, *p* < 0.001), and *R*_inc_ (*F* (9, 171) = 4.36, *p* < 0.001). Simple effects test revealed a significant difference between the first and subsequent trials in these TI parameters (*p* < 0.05; see Table 1 for details). More specifically, *I*_max_, *AUC*, and *R*_inc_ were significantly lower for the first trial than for the subsequent trials, and *T*_max_ was significantly higher for the first trial than for the subsequent trials.

### 3.2. Comparison of TI Curves among Trials

The inter-participant averages of TI curves of retronasal aroma are shown in Figure 3. Two-way repeated measures ANOVA revealed significant main effects of the trial number (*F* (9, 216) = 5.92, *p* < 0.001) and time (*F* (29, 696) = 63.19, *p* < 0.001), and a significant interaction between trial number and time (9*F* (261, 6264) = 4.75, *p* < 0.001). Results of simple effects tests for interaction revealed significant simple main effects of trial number in 12 time windows (medians of each time window = 1–15 s, 39–43 s, and 59 s), and significant simple main effects of time in all trial numbers (*p* < 0.05). Multiple comparisons of paired trials for the significant simple main effects of trial number in each time window, performed using the Ryan method, revealed significant differences between the first and subsequent trials in nine time windows (medians = 1–15 s and 39 s), between the second and subsequent trials in three time windows (medians = 1–5 s), and between the third and ninth trials in one time window (medians = 3 s) (*p* < 0.05; for further details, see in Table 2).

These results indicated that TI curve of retronasal aroma was significantly lower in the first trial than in the subsequent trials, and that TI curve in several time windows immediately after starting the evaluation was significantly lower in the second trial than in the subsequent trials.

TI curves obtained for 60 s after swallowing oolong tea beverage. We divided the period from 0 s to 60 s after swallowing into 30 windows of 2 s each, and calculated the average intensity in each time window. Two-way repeated measures analysis of variance (ANOVA) of intensity was performed with trial number and time as within-subject factors. This analysis revealed a significant interaction between trial number and time. Simple effects tests for interaction revealed significant simple main effects of trial number in 12 time windows (1–15 s, 39–43 s, and 59 s). Multiple comparisons of paired trials for the significant simple main effects of trial number in each time window revealed significant differences between the first and other trials in nine time windows (1–15 s and 39 s), between the second and other trials excluding the first trial in three time windows (1–5 s), and between the third and ninth trials in one time window (3 s).

### 3.3. Approximation of the TI Curve

In Table 3, we show the coefficients and time constants of the exponential functions fitted to the TI curve in each trial, as well as the goodness of fit, represented by the correlation coefficients between the actual values (TI curve) and the theoretical values (fitted exponential function).

The coefficient of the obtained exponential function was smaller in the first trial than in the subsequent trials, but did not significantly differ among the ten trials. The time constant of the fitted exponential function was slightly larger in the second through fourth trials than in the first and the fifth through tenth trials, but did not differ among the ten trials. The average goodness of fit in the first trial was less than 0.7, reflecting a moderate relationship [66]. Average values of goodness of fit in the second through tenth trials were greater than 0.8, reflecting strong or very strong relationships [66]. In regard to goodness of fit, one-way repeated measures ANOVA revealed a significant main effect of the trial number (*F* (9, 216) = 5.23, *p* < 0.001). Multiple comparisons of paired trials for the significant simple main effect of trial number, performed using the Ryan method, revealed significant differences between the first and subsequent trials (*p* < 0.05). More specifically, goodness of fit of the fitted exponential function was significantly lower for the first trial than for the subsequent trials.

## 4. Discussion

### 4.1. Temporal Change of Retronasal Aroma Intensity

In this study, participants continuously evaluated the intensity of retronasal aroma after swallowing oolong tea beverages over ten trials. Based on the data acquired, six types of TI parameters and TI curves were compared among these trials. As a result of the changes in olfactory sensitivity that occur while a participant is sipping a beverage, olfactory sensitivity was significantly higher in the first trial than in the subsequent trials. Additionally, based on the results of the TI curve, olfactory sensitivity between beginning the evaluation and achieving maximum intensity was significantly higher in the second trial than in the subsequent trials, and olfactory sensitivity during the period from the time of maximum intensity to the end of the evaluation in the second to tenth trials did not decrease with repetition. These results were inconsistent with results of previous studies that observed gustatory adaptation in multi-sip single-point evaluation of taste quality [60,61,62]. Instead, we consider that these results were consistent with those of Gotow and colleagues [42], who reported that the experience of consuming certain foods might improve olfactory sensitivity for the retronasal aroma of the food. The consumption experience on which Gotow and colleagues focused was related to dietary habits formed over a relatively long period of time. On the other hand, in this study, we focused on short-term consumption experience, such as what occurs while consumers sip a cup of oolong tea beverage. Based on the above, we concluded that perception of retronasal aroma changes over the course of such a short-term consumption experience.

Regarding for TI parameters, *I*_max_, *T*_max_, *R*_inc_, and *AUC* differed significantly between the first and the subsequent trials. *I*_max_, *T*_max_, and *R*_inc_ were calculated on the basis of the TI curve obtained between the start of the evaluation to the time when retronasal aroma reached maximum intensity. These results suggest that short-term consumption experiences are reflected in retronasal aroma intensity, which is perceived especially immediately after foods are swallowed. Conversely, temporal changes in retronasal aroma intensity after reaching maximum intensity (i.e., *D*_plateau_, and *R*_dec_) may not be significantly affected by short-term consumption experiences. Distel and colleagues [67] reported a significantly positive correlation between familiarity of an aroma and its intensity. Mochizuki-Kawai and colleagues [68] measured reaction time for aroma detection using four types of aromatic substances. They reported that participants detected the aroma with which they were most familiar significantly faster than the aroma with which they were least familiar. The term “detection” generally refers to perception of the presence of an aroma [69] or a change in the olfactory environment [70]. However, when we refer to the results of Mochizuki-Kawai and colleagues in the context of perceiving maximum intensity, we speculate that intake of oolong tea beverage in the first trial may have increased the participant’s familiarity with its aroma, causing the maximum intensity in subsequent trials to increase, and the time required to reach maximum intensity to shorten.

### 4.2. Role as a Warm-Up Sample

In this study, before starting the first trial, the participant confirmed the instructions with the experimenter. In other words, the participant did not experience an exercise trial. The first trial was the first time that they swallowed beverage and reported their intensity of retronasal aroma using the evaluation system. Accordingly, the sample presented in the first trial could be regarded as a warm-up sample, i.e., this means a food sample that is presented to a participant before they evaluate the test samples [71]. There are the three purposes for presenting warm-up sample [71,72]: First, to encourage self-calibration of the evaluation by comparing the individual response of each participant with the consensual response of all participants [73,74,75]; second, for use as a reference sample for the evaluation [76]; and third, to experience the evaluation under conditions similar to those of test trials [77]. In this context, the first trial in this study was conducted to accomplish the third purpose.

Some sensory evaluations reported that the use of a warm-up sample improves the perceptual sensitivity of the participant [78,79,80]. Gotow and colleagues [43] investigated the effect of a warm-up sample in TI evaluation of retronasal aroma and bitterness after swallowing coffee beverages. Half of the participants continuously evaluated retronasal aroma intensity over four trials in the first session, and bitter intensity over four trials in the second session. The remaining half of participants continuously evaluated bitterness intensity over four trials in the first session, and bitterness intensity over four trials in the second session. Participants rested for approximately five minutes between trials. As in this study, no exercise trial was arranged prior to the test trials. Their results demonstrated that when the participant continuously evaluated retronasal aroma intensity in the first and second sessions and bitterness intensity in the first session, TI curve was significantly lower in the first trial than in the subsequent trials. Accordingly, the results of this study reproduced the effect of a warm-up sample in TI evaluation of retronasal aroma, as observed in the previous study [43].

Lawless and Heymann [35] reported that the use of a warm-up sample exerted some stabilizing effect on the sensory evaluation. Consistent with this, the results of this study demonstrated that TI curves did not differ among the second to tenth trials, although the TI curve from the time when participant started the evaluation to the time when they perceived maximum intensity was significantly lower in the second trial than in the subsequent trials. We consider that TI curves among the second to tenth trials might have been almost similar because the sample presented in the first trial served as a warm-up sample.

As described above, TI curve during the time from starting the evaluation to reaching maximum intensity was significantly lower in the second trial than in the subsequent trials. In other words, we inferred that sample presented in the second trial might also function as a warm-up sample, although it did alter the TI curve less drastically than the sample presented in the first trial. Gotow and colleagues [43] reported that the effect of a warm-up sample was observed even when time-intensity evaluation of retronasal aroma was arranged in not only the first session, but the second session after performing TI evaluation of bitterness in the first session. Some previous studies, in which psychological experiments were performed using taste solutions containing aromas, reported that it was more difficult for participants to detect and identify olfactory than gustatory components because they directed voluntarily their attention to tongue [36,37]. Based on those studies, because participants needed some practice to direct their attention to retronasal aroma, TI evaluation only in the first trial might not provide sufficient exercise. However, the results implied that similar TI curves might be obtained, because lack of practice disappeared within approximately 10 s after starting the evaluation in the second trial. Furthermore, time period during which intensity differed significantly between the first and subsequent trials was less than 20 s after starting the evaluation. Once the participant could direct their attention suitably to retronasal aroma in each trial, the intensity should always depict similar traces independently of trial number.

### 4.3. Improvement of Olfactory Sensitivity by Short-Term Consumption Experience

In this study, we calculated the fitted exponential function of the TI curve. The goodness of fit of the exponential model to the TI curve was significantly lower in the first trial than in subsequent trials. This result revealed that performance in the first trial was not only poor in terms of perceptual sensitivity, but inconsistent with the hypothesis that intensity decreases exponentially with time. The coefficient of the fitted exponential function, corresponding to the maximum intensity, did not differ significantly among ten trials. This was not consistent with the results of the TI parameter *T*_max_. The inconsistency between the coefficient and *T*_max_ may have been affected by the low goodness of fit of the TI curve in the first trial. In addition, the time constant of the fitted exponential function did not differ among ten trials. This was consistent with the result of the TI parameter *R*_dec_. However, the time constant was slightly larger in the second through fourth trials than in the first and the fifth through tenth trials. The higher the value of the time constant, the faster the decay of intensity. These results implied that perceptual sensitivity to retronasal aroma was improved by a short-term consumption experience, although TI curves did not drastically change from the second to the tenth trials.

We propose the following three hypotheses to explain how short-term consumption experience affected the TI curve of retronasal aroma. First, aromatic substances contained in oolong tea beverage may remain in the olfactory mucosa, oral cavity, and esophagus. Many previous studies reported that intensity of aroma depends on concentration [81,82,83,84].

Second, the participants might have been able to easily predict what kind of aroma was perceived. Distel and Hudson [85] divided participants into two groups, and then performed an intensity evaluation of everyday odors. One group was presented odors with a name, and they then evaluated the intensity of odor and the suitability of the name. Another group was presented odors without a name, and they then evaluated the intensity of the odor and identified the name. The results demonstrated that participants reported the highest intensity when the odor name provided by the experimenter matched with the participant’s perception. Oolong tea is a familiar beverage to Japanese consumers [47], but its aroma differs among products [86]. In this study, participants measured the oolong tea beverage at the beginning of each trial. Therefore, we inferred that participants could realize relatively easily that they were sipping the same beverage repetitively. Such repetitive intake might reinforce the relationship between the cognitive representation of the aroma of oolong tea beverage and the practical experience of olfactory perception.

Third, it is possible that exposure to the aroma of oolong tea beverage changed brain activity in the olfactory-related area. Veldhuizen and Small [87], who identified brain areas related to attention using functional magnetic resonance imaging, reported that brain activity increased in piriform cortex, ventral insula, (para)hippocampal gyrus, mediodorsal thalamus, substantia nigra, cerebellum, anterior insula, and frontal operculum when the participant was instructed to direct their attention to aroma. Of these brain areas, the piriform cortex is specialized for processing of olfactory information. When a participant was instructed to direct their attention to taste quality, brain activity in piriform cortex did not increase. Furthermore, Li and colleagues [88], who investigated the relationship between experience-induce olfactory perceptual learning and plasticity of the brain, reported that brain activity increased in piriform cortex and orbitofrontal cortex upon exposure to aroma. Based on these previous studies, we postulated that neural representation in olfactory-related brain areas might accelerate rapidly over the relatively short period of time required for a participant to sip a cup of beverage. We intend to examine the validity of these three hypotheses in the near future.

### 4.4. Current Limitation and Future Issues

In this study, participants evaluated only one sensory attribute of food (i.e., retronasal aroma of oolong tea beverage). When a participant evaluates only a specific sensory attribute rather than evaluating multiple sensory attributes, a “halo damping effect” [89] may occur [17,22]. More specifically, if only a specific sensory attribute is evaluated, the evaluation value of a given sensory attribute may change due to other sensory attributes [90,91]. Therefore, we cannot exclude the possibility that the halo damping effect occurred in this study. Gotow and colleagues [34,42,43], who employed TI evaluation of bitterness and retronasal aroma of coffee beverages using a within-subjects design, adopted the same procedure used in this study; i.e., participants were asked to evaluate a single sensory attribute per trial. However, in order to reduce the occurrence of the halo damping effect as much as possible, participants were informed about all sensory attributes to be evaluated before beginning the TI evaluation, and they were instructed to emphasize a specific sensory attribute to be evaluated in each trial. In the future, we should investigate whether we can obtain results similar to those of this study even when employing TI evaluation of multiple sensory attributes of oolong tea (e.g., bitterness, umami, and astringency) using an inter-subjects design and the same devices used in previous studies.

Oolong tea contains multiple types of bioactive compounds, such as catechin and caffeine [92]. Xu and colleagues [55] measured catechin concentration and evaluated astringency in oolong tea extracted with four types of water (i.e., purified water, mineral water, mountain spring water, and tap water from Hangzhou). Catechin concentration was significantly lower when oolong tea was brewed with mineral or tap water than with purified or spring water, indicating that oolong tea brewed in water with higher conductivity (i.e., water containing a lot of minerals) has a lower catechin concentration. Additionally, astringency was almost equal between oolong tea brewed with mineral or tap water, but was significantly higher for tea brewed with mineral water than with purified or spring water. Based on the results of Xu and colleagues [55], it is possible that the ions in mineral water may interact with the bioactive components in oolong tea, thereby affecting the astringency of the resultant beverage. In this study, participants cleaned their mouth using mineral water. The evaluation of the astringency of oolong tea may change depending not only on the type of water used for brewing the tea, but also on the type of water used for oral cleaning. We will address this hypothesis in future work.

## 5. Implication

Sensory evaluation is essential in food industries to routinely monitor the quality of beverages and to ensure that the beverage products are acceptable to customers [93]. TI evaluation for measuring temporal change in a specific sensory attribute is a common method in time-based sensory evaluation of beverage [2]. The results of this study, which performed multi-sip TI evaluation without training trials, suggest that untrained panelists’ olfactory perception differed remarkably between the first and subsequent sips of drinking. This finding demonstrates the significance for food industries to perform sensory evaluation after understanding the specificity of olfactory perception in the first sip of drinking.

## 6. Conclusions

In this study, we performed multi-sip TI evaluation of retronasal aroma. In each of ten consecutive trials, after a participant swallowed oolong tea beverage, they continuously evaluated intensity of retronasal aroma over 60 s. We compared six types of TI parameters (*I*_max_, *T*_max_, *AUC*, *D*_plateau_, *R*_inc_, and *R*_dec_) and TI curves among ten trials, and approximated each TI curve with an exponential model, using the least-squares method. Some TI parameters (i.e., *I*_max_, *T*_max_, *AUC*, and *R*_inc_) differed significantly among the first and subsequent trials. TI curve was significantly lower in the first trial than in the subsequent trials, and TI curve during the time from staring the evaluation to reaching maximum intensity was significantly lower in the second trial than in the subsequent trials. The time constant of the fitted exponential function revealed that the decay of retronasal aroma intensity was slightly faster in the second through fourth trials than in the first and the fifth through tenth trials. These results implied that olfaction might not adapt, but instead become more perceptive while a consumer sips a cup of beverage.

## Figures and Tables

**Table 1:** Multiple comparisons of paired trials for significant simple main effects of trial number for each TI parameter.

Parameter	Trial									
	1st	2nd	3rd	4th	5th	6th	7th	8th	9th	10th
*I*_max_	3.71 ^a^	4.13 ^b^	4.29 ^b^	4.21 ^b^	4.25 ^b^	4.22 ^b^	4.26 ^b^	4.29 ^b^	4.32 ^b^	4.20 ^b^
	(1.00)	(0.68)	(0.64)	(0.62)	(0.66)	(0.68)	(0.67)	(0.62)	(0.52)	(0.74)
*T*_max_	14.88 ^a^	7.92 ^b^	5.98 ^b^	5.97 ^b^	5.22 ^b^	7.10 ^b^	4.60 ^b^	8.36 ^b^	3.83 ^b^	5.95 ^b^
	(12.83)	(9.40)	(6.74)	(5.76)	(3.65)	(9.42)	(1.82)	(12.44)	(1.97)	(5.15)
*AUC*	110.16 ^a^	133.08 ^b^	143.25 ^b^	142.57 ^b^	145.59 ^b^	147.26 ^b^	149.78 ^b^	147.80 ^b^	151.45 ^b^	147.02 ^b^
	(43.20)	(42.82)	(41.31)	(46.43)	(50.76)	(42.87)	(44.59)	(50.85)	(50.38)	(52.72)
*D*_plateau_	8.79 ^a^	8.83 ^a^	9.61 ^a^	12.66 ^a^	12.25 ^a^	11.73 ^a^	8.74 ^a^	9.67 ^a^	7.87 ^a^	7.52 ^a^
	(8.03)	(6.91)	(6.94)	(14.42)	(12.93)	(12.57)	(8.68)	(8.52)	(7.44)	(5.19)
*R*_inc_	1.13 ^a^	2.28 ^b^	2.33 ^b^	2.28 ^b^	2.19 ^b^	2.59 ^b^	2.10 ^b^	2.34 ^b^	2.90 ^b^	2.87 ^b^
	(1.13)	(1.64)	(1.39)	(1.22)	(1.31)	(1.92)	(1.50)	(1.65)	(1.91)	(2.42)
*R*_dec_	−0.09 ^a^	−0.07 ^a^	−0.07 ^a^	−0.11 ^a^	−0.15 ^a^	−0.12 ^a^	−0.06 ^a^	−0.06 ^a^	−0.06 ^a^	−0.06 ^a^
	(0.06)	(0.04)	(0.03)	(0.22)	(0.40)	(0.30)	(0.02)	(0.03)	(0.03)	(0.04)

Average values of TI parameters with standard deviation in parentheses are shown for each trial. For each parameter, trial numbers with different alphabets differed significantly (*p* < 0.05). The unit used for *T*_max_ and *D*_plateau_ is second. *AUC*: area under the TI curve; *I*_max_: maximum intensity; *T*_max_: time point at which intensity reached the maximum value; *AUC*: area under the TI curve; *D*_plateau_: duration between *T*_90%start_ and *T*_90%end_; *R*_inc_: rate of intensity increase between *T*_5%start_ and *T*_90%start_; *R*_dec_: rate of intensity decrease between *T*_90%end_ and *T*_5%end_.

**Table 2:** Multiple comparisons of paired trials for the significant simple main effects of trial number in each time window.

Time Window	Trial									
(in seconds)	1st	2nd	3rd	4th	5th	6th	7th	8th	9th	10th
1	0.01 ^a^	0.43 ^ab^	0.75 ^bc^	0.84 ^bc^	0.90 ^bc^	0.97 ^c^	0.94 ^c^	0.95 ^c^	1.14 ^c^	0.98 ^c^
	(0.22)	(1.02)	(1.18)	(1.12)	(0.19)	(1.28)	(1.30)	(1.22)	(1.35)	(1.34)
3	0.65 ^a^	2.16 ^b^	2.76 ^c^	2.82 ^cd^	2.82 ^cd^	2.87 ^cd^	2.85 ^cd^	3.16 ^cd^	3.29 ^d^	2.83 ^cd^
	(1.06)	(1.69)	(1.63)	(1.65)	(1.47)	(1.38)	(1.50)	(1.24)	(1.18)	(1.48)
5	1.39 ^a^	3.17 ^b^	3.62 ^bc^	3.43 ^bc^	3.55 ^bc^	3.60 ^bc^	3.64 ^bc^	3.65 ^bc^	3.77 ^c^	3.61 ^bc^
	(1.56)	(1.50)	(1.24)	(1.34)	(1.20)	(1.03)	(1.03)	(1.17)	(0.89)	(1.12)
7	2.06 ^a^	3.45 ^b^	3.69 ^b^	3.51 ^b^	3.62 ^b^	3.63 ^b^	3.62 ^b^	3.54 ^b^	3.68 ^b^	3.59 ^b^
	(1.58)	(1.30)	(1.02)	(1.27)	(1.11)	(0.97)	(1.06)	(1.23)	(0.97)	(1.11)
9	2.49 ^a^	3.41 ^b^	3.71 ^b^	3.53 ^b^	3.64 ^b^	3.67 ^b^	3.62 ^b^	3.58 ^b^	3.69 ^b^	3.61 ^b^
	(1.54)	(1.15)	(0.82)	(1.06)	(0.82)	(0.76)	(0.86)	(0.91)	(0.70)	(0.80)
11	2.69 ^a^	3.31 ^b^	3.51 ^b^	3.39 ^b^	3.38 ^b^	3.36 ^b^	3.40 ^b^	3.37 ^b^	3.47 ^b^	3.41 ^b^
	(1.55)	(1.05)	(1.05)	(1.11)	(1.08)	(1.09)	(1.08)	(1.07)	(1.07)	(1.16)
13	2.58 ^a^	3.19 ^b^	3.49 ^b^	3.39 ^b^	3.36 ^b^	3.43 ^b^	3.43 ^b^	3.36 ^b^	3.49 ^b^	3.46 ^b^
	(1.57)	(1.04)	(0.77)	(0.84)	(0.80)	(0.75)	(0.83)	(0.86)	(0.81)	(0.84)
15	2.56 ^a^	3.12 ^b^	3.32 ^b^	3.24 ^b^	3.17 ^b^	3.28 ^b^	3.23 ^b^	3.29 ^b^	3.27 ^b^	3.26 ^b^
	(1.37)	(1.05)	(0.85)	(0.94)	(0.85)	(0.88)	(0.91)	(0.87)	(0.92)	(0.93)
39	1.74 ^a^	1.97 ^ab^	2.18 ^ab^	2.03 ^ab^	2.17 ^ab^	2.20 ^ab^	2.33 ^b^	2.16 ^ab^	2.26 ^ab^	2.25 ^ab^
	(0.01)	(1.00)	(0.88)	(0.96)	(1.02)	(0.84)	(0.82)	(1.07)	(1.00)	(1.05)

Inter-participant average of intensity with standard deviation in parentheses only in time windows in which differed significantly between paired trials are shown. Values of each time window are medians (e.g., 1 second means the time window from 0–2 s). For each time window, trials marked with different letters differed significantly (*p* < 0.05).

**Table 3:** Coefficient, time constant, and correlation coefficient of fitted functions for inter-participant average of TI curve.

Parameter	Trial									
	1st	2nd	3rd	4th	5th	6th	7th	8th	9th	10th
Coefficient	4.250 ^a^	5.145 ^a^	5.048 ^a^	4.816 ^a^	4.602 ^a^	4.561 ^a^	4.405 ^a^	4.712 ^a^	4.587 ^a^	4.708 ^a^
	(1.892)	(1.948)	(1.635)	(1.623)	(1.491)	(1.278)	(1.209)	(1.579)	(1.405)	(1.661)
Time constant	0.022 ^a^	0.028 ^a^	0.024 ^a^	0.024 ^a^	0.022 ^a^	0.021 ^a^	0.019 ^a^	0.023 ^a^	0.020 ^a^	0.022 ^a^
	(0.019)	(0.023)	(0.014)	(0.016)	(0.017)	(0.012)	(0.012)	(0.021)	(0.015)	(0.020)
Goodness of fit	0.662 ^a^	0.867 ^b^	0.908 ^b^	0.900 ^b^	0.848 ^b^	0.894 ^b^	0.894 ^b^	0.839 ^b^	0.906 ^b^	0.895 ^b^
	(0.395)	(0.234)	(0.122)	(0.191)	(0.255)	(0.208)	(0.171)	(0.290)	(0.178)	(0.200)

In the exponential function *y* = *A* × exp (−*Bt*), *A* and *B* are coefficient and time constant, respectively. Goodness of fit is represented by Pearson’s product–moment correlation coefficient calculated between actual values (TI curve) and theoretical values (fitted exponential function). Average values of each parameter with standard deviation in parentheses are shown. For each parameter, trials marked with different letters differed significantly (*p* < 0.05).
